# Spontaneous Immunity Against the Receptor Tyrosine Kinase ROR1 in Patients with Chronic Lymphocytic Leukemia

**DOI:** 10.1371/journal.pone.0142310

**Published:** 2015-11-12

**Authors:** Mohammad Hojjat-Farsangi, Mahmood Jeddi-Tehrani, Amir Hossein Daneshmanesh, Fariba Mozaffari, Ali Moshfegh, Lotta Hansson, Seyed Mohsen Razavi, Ramazan Ali Sharifian, Hodjattallah Rabbani, Anders Österborg, Håkan Mellstedt, Fazel Shokri

**Affiliations:** 1 Department of Oncology-Pathology, Immune and Gene Therapy Lab, Cancer Center Karolinska (CCK), Karolinska University Hospital Solna and Karolinska Institute, Stockholm, Sweden; 2 Department of Immunology, School of Public Health, Tehran University of Medical Sciences, Tehran, Iran; 3 Monoclonal Antibody Research Center, Avicenna Research Institute, ACECR, Tehran, Iran; 4 Department of Hematology-Oncology, Karolinska University Hospital Solna, Stockholm, Sweden; 5 Clinic of Hematology and Oncology, Firozgar Hospital, Faculty of Medicine, Iran University of Medical Sciences, Tehran, Iran; 6 Clinic of Hematology and Oncology, Vali-Asr Hospital, Faculty of Medicine, Tehran University of Medical Sciences, Tehran, Iran; University of Manitoba, CANADA

## Abstract

**Background:**

ROR1 is a receptor tyrosine kinase expressed in chronic lymphocytic leukemia (CLL) and several other malignancies but absent in most adult normal tissues. ROR1 is considered an onco-fetal antigen. In the present study we analysed spontaneous humoral and cellular immunity against ROR1 in CLL patients.

**Materials and Methods:**

Antibodies against ROR1 were analysed in 23 patients and 20 healthy donors by ELISA and Western blot. Purified serum IgG from patients was tested for cytotoxicity against CLL cells using the MTT viability assay. A cellular immune response against ROR1 derived HLA-A2 restricted 9 aa and 16 aa long peptides were analysed using peptide loaded dendritic cells co-cultured with autologous T cells from CLL patients (n = 9) and healthy donors (n = 6). IFN-γ, IL-5 and IL-17A-secreting T cells were assessed by ELISPOT and a proliferative response using a H3-thymidine incorporation assay.

**Results:**

The majority of CLL patients had antibodies against ROR1. Significantly higher titers of anti-ROR1 antibodies were noted in patients with non-progressive as compared to progressive disease. The extracellular membrane-close ROR1 KNG domain seemed to be an immunodominant epitope. Ten patients with high titers of anti-ROR1 binding antibodies were tested for cytotoxicity. Five of those had cytotoxic anti-ROR1 antibodies against CLL cells. ROR1-specific IFN-γ and IL-17A producing T cells could be detected in CLL patients, preferentially in non-progressive as compared to patients with progressive disease (p<0.05).

**Conclusion:**

ROR1 seemed to spontaneously induce a humoral as well as a T cell response in CLL patients. The data support the notion that ROR1 might be a specific neo-antigen and may serve as a target for immunotherapy.

## Introduction

Chronic lymphocytic leukemia (CLL) is the most prevalent leukemia in the West with an accumulation of clonal CD5^+^/CD19^+^/CD23^+^ B cells in the blood, bone marrow, lymph nodes, and spleen. The clinical outcome is highly variable but most patients develop symptomatic disease and die from causes related to the disease [1].

Gene profiling of CLL cells has revealed up- and down-regulations of hundreds of genes with various chromosomal localizations [2,3]. The receptor tyrosine kinase (RTK) gene *ROR1* was upregulated 45-folds in CLL cells compared to normal memory B cells [2]. The human *ROR1* gene is located to the chromosomal region 1p31.3 and a member of the RTK family related to muscle specific kinase and Trk neurotrophin receptors [4,5]. The coding region is 2814 bp and the protein consists of 937 amino acids with a predicted size of 105 kDa. The ROR1 molecule has an extracellular part, including an Ig-like (Ig), cysteine-rich (CRD) and kringle (KNG) domain as well as an intracellular part consisting of tyrosine kinase and proline-rich domains [6,7]. The ROR1 protein is expressed on the surface of CLL cells and several other B cell malignancies as well as in solid tumors, but not on normal B cells and most other adult normal tissues [6,8–10].

ROR1 seems to fulfill several criteria for being a tumor associated antigen (TAA) [6] and might as such be recognized by the immune system. Fukuda et al [10] showed the induction of ROR1-specific antibodies in CLL patients immunized with CLL cells transduced with CD154 expressed in an adenovirus vector, Ad-CD154. The antibodies inhibited Wnt5a dependent proliferation of CLL cells induced by Wnt5a. Immunization of ROR1 transgenic mice with ROR1 peptides induced anti-ROR1 antibodies, which inhibited engraftment of human ROR1^+^ CLL cells [11].

In the present study, we analysed spontaneously induced ROR1 antibodies as well as a cellular immune response in CLL patients. A ROR1 specific spontaneous immune response may support the assumption of ROR1 as a tumor neo-antigen.

## Materials and Methods

### Patients and controls

Twenty three CLL patients were analyses for anti-ROR1 antibodies. Fifteen of those were in a non-progressive phase while 8 had progressive disease. Sera from 20 age-matched healthy donors were used as controls. Nine CLL patients (HLA-A2^+^) and 6 HLA-age matched control donors were evaluated for a T-cell response against ROR1 derived peptides. Six of those patients had non-progressive disease at the time of testing while 3 were in a progressive phase. The diagnosis of CLL was determined as previously described [9]. Patients were considered to have progressive disease if the following criteria were met: progression during the preceding 3 months in disease-related anemia (hemoglobin <100g/l), thrombocytopenia (<100×10^9^/l) and/or an increase in spleen/liver/lymph-node size and/or more than a 2-fold increase in the blood lymphocyte count, if not the patients were considered non-progressive [9]. HLA-A alleles were determined by genomic DNA typing using the SSP-PCR method as previously described [12]. Written informed consent was obtained from all patients and controls. The study was approved by the Regional Ethics Committee at the Karolinska Institute, Stockholm, Sweden (www.epn.se).

### Isolation of blood mononuclear cells

Peripheral blood mononuclear cells (PBMC) from CLL patients and normal donors were separated from peripheral blood using Histopaque (Sigma-Aldrich, St Louis, MO, USA) density-gradient centrifugation as described [9].

### Serum anti-ROR1 antibodies

#### Detection of anti-ROR1 antibodies by Western blot

A full length recombinant ROR1 protein (OriGene Technologies, Rockville, MD, USA) and a recombinant KNG domain (Protein Abnova Corporation, Taipei, Taiwan) respectively as well as immunoprecipitated ROR1 protein from CLL cell lysates were used in Western blot.

For immunoprecipitation, two hundred ul of lysis buffer containing 1% Triton X-100, 50 mM Tris-HC1, pH 7.4, 150 mM NaCl, 5 mM EDTA, and 1% protease inhibitor cocktail (Sigma-Aldrich) was added to 5×10^6^ CLL cells. Cells were incubated on ice for 30 min with vortexing. The concentration of the protein lysate was measured by BCA Protein Assay Kit (Thermo Scientific, IL, USA). Five hundred ug of the cell lysate in 200 ul of PBS buffer was precleared twice with 100 ul of protein G and protein A agarose (Calbiochem, Darmstadt, Germany) at 4°C for 2 h. The supernatant was collected and incubated with 100 ul of protein G, protein A agarose and 3 ug of a goat anti-human ROR1 polyclonal antibody (against the full length ROR1 protein) (R&D system; Minneapolis, MA, USA), an anti-CD20 MAb (monoclonal antibody) or an isotype control MAb (BD Biosciences, San Jose, CA, USA) overnight at 4°C. Finally, protein G and protein A agarose were washed 3 times with PBS and suspended in 100 ul of PBS for further analysis in Western blot.

One ug of each recombinant proteins as well as twenty ul of immunoprecipitated ROR1 from CLL cells was run on a 10% Bis-Tris SDS-PAGE gel (Invitrogen, Carlsbad, CA, USA) at 100 V for 4 h under reducing conditions. After electrophoresis, resolved proteins were transferred onto Immobilon-PVDF membranes (Millipore AB, Stockholm, Sweden) in a mini Transblot cell (Invitrogen). The membranes were blocked for 2 h at room temperature with 5% non-fat milk in PBS with 0.1% Tween 20 (PBS-T). Filters were incubated with sera (1:50) from CLL patients and control donors in blocking buffer overnight at 4°C. After extensive washing with PBS-T, filters were incubated with goat anti-human Ig conjugated with HRP (Dako, Glostrup, Denmark) for 1 h at room temperature followed by washing and developed with advanced ECL chemiluminescence detection system (GE Healthcare, Uppsala, Sweden). The goat anti-ROR1 antibody, the anti-CD20 MAb and the isotype control MAb were also used as controls.

#### Detection of anti-ROR1 antibodies by ELISA

The recombinant full length ROR1 protein and the recombinant ROR1 KNG protein were used in ELISA as antigens for detection of anti-ROR1 and anti-KNG ROR1 antibodies, respectively. A recombinant carcinoembryonic antigen (CEA) protein (Protein Sciences Corp. Meriden, Mass, USA) was used as a control. Ninety-six wells ELISA plates were coated with 100 ul of the recombinant proteins dissolved in PBS (2.8 ug/ml) and incubated at 4°C overnight. The plates were washed 5 times with PBS-T for 5 min. One hundred ul of non-fat milk (5%) was added to each well and the plates were incubated at room temperature for 3 h. The wells were washed and 100 ul of sera from patients and control donors in PBS (1:50) was added to each well in duplicates. A human anti-ROR1 antibody (clone G03) (Kancera AB, Stockholm, Sweden) was used as standard antibody for quantitative measurement of anti-ROR1 antibodies. The plates were incubated at room temperature for 1 h and washed. A goat anti-human Ig conjugated with HRP (1:1000) (Avicenna Research Center, Tehran, Iran) was added and the plates were incubated at room temperature for 1 h and washed 5 times. Finally, 100 ul of tetramethylbenzidine (TMB) substrate (R&D) was added to each well. The plates were incubated at room temperature in the dark. After 7 min, the reaction was stopped by adding 30 ul of stopping solution (0.16 M sulfuric acid). Optical density (OD) was measured at 450 nanometer by an ELISA reader. The concentration of anti-ROR1 and anti-KNG antibodies was estimated using a standard curve.

### Cell viability testing (cytotoxicity)

The effect of sera on the viability (cytotoxicity) of leukemic cells was measured using the isolated serum IgG fraction from 10 CLL patients and 10 control donors [HiTrap Protein G HP columns (GE Health Care) [13].

The isolated IgG fraction from CLL patients were also adsorbed by affinity column chromatography using the full length recombinant ROR1 protein immobilized on periodate activated Sepharose to deplete ROR1 antibodies. Briefly, Sepharose 4B (GE Health Care) was washed with 10–20 volumes of water. One volume of Sepharose 4B was mixed with two volumes of sodium periodate in water (20 mg/ml) and incubated for 2 h at room temperature on a rotator. After incubation, Sepharose 4B was washed with 5 volumes of water and 3 volumes of 100 mM sodium borate, pH 9.0–9.4. One volume of the recombinant full length ROR1 protein (1 mg/ml) in buffer (pH 9.0–9.4) was added to one volume of Sepharose 4B and incubated on a rotator for 3 h. Sepharose was added to a micropipette. The column was washed twice with two volumes of 100 mM sodium borate (pH 8.0). Equal volumes of sodium borohydride (3 mg/ml) in 100 mM sodium borate (pH 8.0) was added to the Sepharose and incubated on the rotator for 5 min and washed repeatedly with sodium borate. Sepharose was then blocked by washing three times with 2 volumes of 100 mM ethanolamine, 0.5 M NaCl, (pH 8.0). Finally, the column was washed with PBS buffer 200 ul (1 mg/ml) of the isolated IgG was then added to the column and incubated at room temperature for 2 h. Total IgG was then collected after adsorption.

Serum anti-ROR1 antibodies were adsorbed of each CLL patient by incubating 1 ml serum with 1 mg lysate from ROR1 expressing CLL cells at 4°C for overnight on a shaker. The cells were centrifuged at 30000g, supernatants collected, and concentrated forty times.

The sera were tested for cell viability using leukemic CLL cells and normal PBMC, as target cells in MTT assay in triplicates as previously described [13]. Briefly, 10^4^ CLL cells or normal PBMC were incubated in 200 ul RPMI-1640 (Invitrogen) containing 10% FBS and 10 ug/ml of IgG from CLL patients before and after adsorption as well as IgG from control donors. Medium alone and untreated cells were used as controls. Plates were incubated at 37°C for 24 h. One hundred ul of MTT solution (5 mg/ml) (Sigma-Aldrich) in PBS was added. The cells were incubated for a further 4 h at 37°C. Fifty ul of stop solution (4mM HCl, 0.1% NP-40 in isopropanol) was added and incubated for 2 h at 37°C.Cytotoxicity (%) was calculated as described [13].

### Generation of dendritic cells

Dendritic cells (DC) were generated from blood CD14^+^ cells of control donors and CLL patients as previously described [14]. Briefly, PBMC were incubated with anti-CD14 coated nanobeads (Miltenyi Biotec, Bergisch Gladbach, Germany). After washing, cells were passed through MidiMACS columns and CD14^+^ cells were separated according to the manufacturer’s instruction. Cells were resuspended in RPMI medium. The purity of CD14^+^ cells (>95%) was determined by flow cytometry. Isolated CD14^+^ cells were cultured with recombinant human (rh) GM-CSF (50 ng/ml) (Peprotech, London, UK) and rhIL-4 (20 ng/ml) (Peprotech) for 5 days. After 4 days of culture, the peptides (10 ug/ml) (see below) were added. For maturation, rhTNF-α (20 ng/ml) (Peprotech) and poly IC (50 ug/ml) (Invitrogen) were added at day 5. The cells were cultured for further 2 days. Harvested cells had the morphology of mature DC with a phenotype of CD3^–^, CD14^–^, CD19^–^, CD80^+^, CD86^+^ and HLA-II^+^ determined by flow cytometry [14].

### Isolation of T cells

T cells were purified from PBMC of control donors and CLL patients as previously described [15]. Briefly, PBMC were washed three times with PBS and passed through nylon wool columns (Polysciences Europe GmbH, Eppelheim, Germany). The effluent cells were enriched by negative selection of T cells using nanobeads (Miltenyi Biotec) according to manufacturer’s instruction. The final purity of T cells (CD3^+^) was >95% as determined by flow cytometry.

### ROR1 peptide design

Four 9 amino acid (aa) long HLA-A2 restricted ROR1 peptides (ROR-p1, p2, p8, p10) and one 16 aa long peptide (ROR-p16) were synthesized based on the ROR1 sequence. Peptides were designed with medium affinity for HLA-A2 and solubility in water. Binding affinity of peptides for HLA-A2 was predicted using two independent HLA peptide binding prediction algorithms, *SYFPEITHI* (http://www.syfpeithi.de) and *BIMAS* (http://www-bimas.cit.nih.gov). Synthesized peptides purchased from Thermo Electron Corporation (GmbH, Ulm, Germany) were of immunograde with >70% purity. A 9 aa long peptide from the influenza virus matrix protein (recall antigen) (aa 58–66; GILGFVFTL) [16], a HIV reverse transcriptase 9 aa long peptide (aa 476–484; ILKEPVHGV) [17] and a 17 aa long mutated Ras peptide (aa 5–21) (GemVax AS, Porsgrunn, Norway) were used as controls (Table 1). All peptides were dissolved in an appropriate solvent at a peptide concentration of 1 mg/ml and stored at -70°C until use.

**Table 1:** ROR1 peptide sequences selected for the T cell assays.

Peptide name	Peptide sequence	Position	
ROR-p1	VATNGKEVV	Ig domain, position 132–140	16
ROR-p2	TMIGTSSHL	CRD domain, position 207–215	22
ROR-p8	SLSASPVSN	intracytoplasmic domain, position 772–780	16
ROR-p10	NKSQKPYKI	intracytoplasmic domain, position 904–912	12
ROR-p16	LQPYYGFSNQEVIEMVRKRQ	intracytoplasmic domain, 690–709	
Positive control	GILGFVFTL	Influenza matrix protein 58–66	30
Negative control	ILKEPVHGV	HIV RT enzyme, position 476–484	30
Negative control	KLVVVGAAGVGKSALTI	p21 Ras peptide, position 5–21	

### Enzyme-linked immunospot assay (ELISPOT)

The frequency of autologous T cells producing IFN-γ, IL-5 and IL-17A in response to the peptides as well as PHA (mitogenic activator) (GIBCO, Stockholm, Sweden) and PPD (recall antigen) (Statens Serum Institute, Copenhagen, Denmark) was determined by ELISPOT as previously described [18]. Briefly, 96-well flat-bottomed PVDF microtiter plates (Millipore) were coated with an anti-human IFN-γ monoclonal antibody (mAb) (clone 1-D1K; Mabtech AB, Stockholm, Sweden), an anti-human IL-5 mAb (clone TRFK5; Mabtech AB) and an anti-human IL-17A mAb (R&D) and incubated at 4°C overnight. After washing, DC (2×10^4^) loaded with the respective peptides were added and cocultured with freshly isolated autologous T cells (2×10^5^) in triplicate at 37°C for 48 h. Biotinylated anti-IFN-γ mAb (clone Mab7-B6-1; Mabtech AB), anti-IL-5 mAb (clone 5A10; Mabtech AB), anti-IL-17A mAb (R&D) and avidin-conjugated alkaline phosphatase (Mabtech AB) were added. BCIP/NBT (Sigma-Aldrich) was used for the colorimetric reaction. Spots were quantitated manually or by an automatic ELISpot reader (Axioplan2; Carl Zeiss Vision, Jena, Germany). The number of spots in cultures incubated with the control peptides were subtracted from the number of spots in ROR1 peptides stimulated cells.

### T cell proliferation assay

The assay has been described in details earlier [15]. Briefly, DC loaded with ROR1 peptides (2×10^4^ cells) were cocultured with autologous T cells (2×10^5^) in 96-well flat-bottomed plates in triplicates, and incubated at 37°C for 5 days in RPMI-1640 medium, containing 10% heat-inactivated human AB^+^ serum, 100 U/ml penicillin and 100 ug/ml streptomycin. One uCi H^3^-thymidine (Amersham Pharmacia Biotech, Uppsala, Sweden) was added to each well and incubated for 18 h at 37°C. Cells were harvested and incorporated radioactivity was measured in a beta-scintillation counter (Wallac 1410 Liquid Scintillation Counter, Pharmacia, Stockholm, Sweden). T cells were also cultured alone or together with unpulsed autologous DC as well as with DC pulsed with control peptides. Results are presented as stimulation index (SI): cpm of cells with test peptides/cpm of cells with control peptides. SI are also shown for cells stimulated with PHA (5 ug/ml) or PPD (2.5 ug/ml).

### Statistical analysis

Statistical analyses were performed applying independent T test, Mann-Whitney U test and the Kruskal-Wallis test using the SPSS statistical package (SPSS Inc., Chicago, IL, USA). A p-value <0.05 was considered statistically significant.

## Results

### Detection of antibodies against ROR1 using immunoprecipitated ROR1 from CLL cells in Western blot

**Fig 1:**
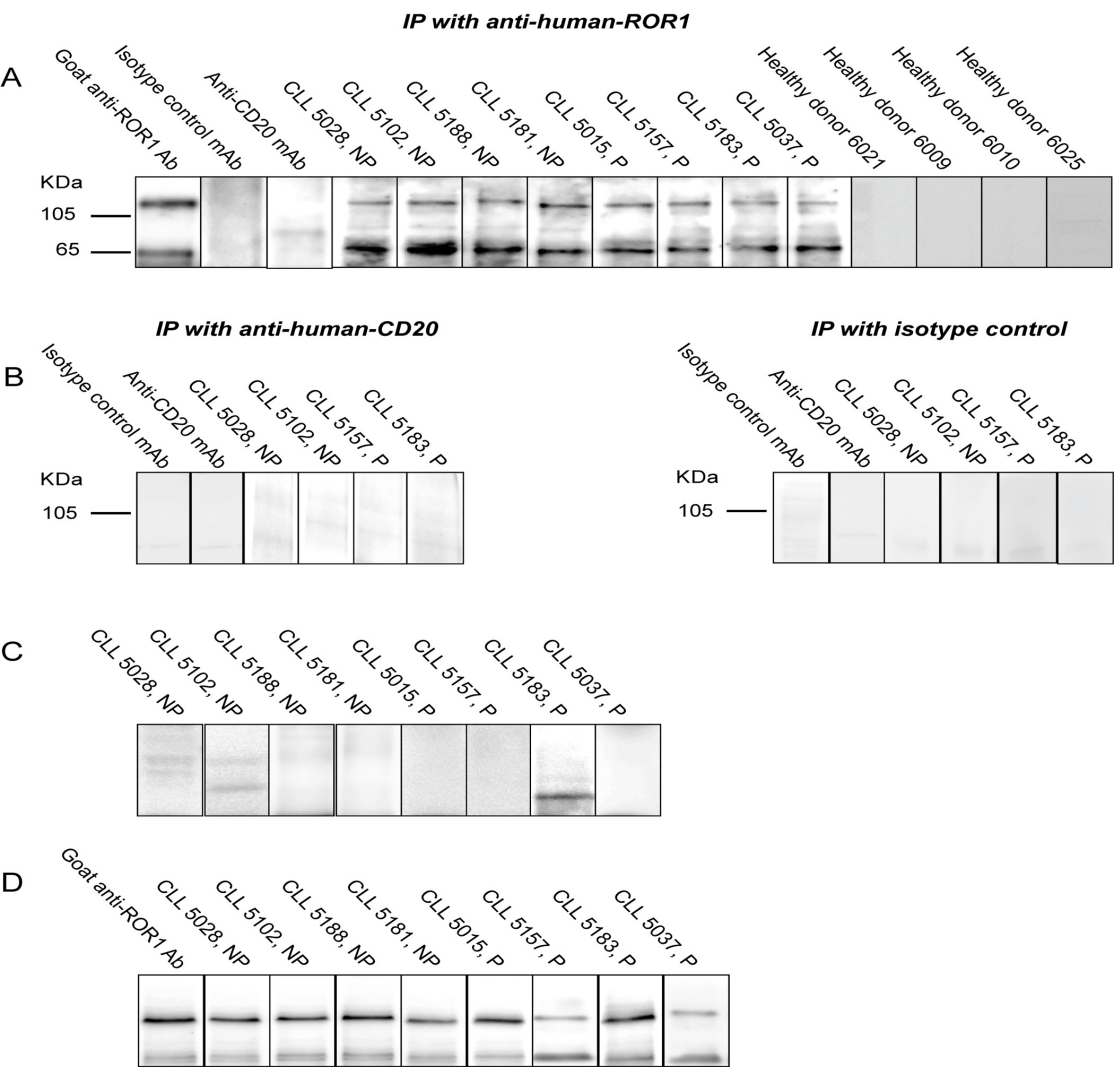
(A) Immunoprecipated (IP) ROR1 from CLL cell lysates probed with serum from 4 non-progressive (NP) and 4 progressive (P) CLL patients as well as 4 control donors. Bands of 105 and 64 kDa could be seen. The blots were also probed with a goat anti-human ROR1 antibody, anti-CD20 MAb and an isotype control MAb. (B) CLL cells immunoprecipitated using anti-CD20 and isotype control MAbs. No ROR1 bands could be detected. (C) Patients’ sera were adsorbed with a pool of CLL cell lysate (n = 10). The 105 and 64 kDa bands disappeared or were significantly reduced. (D) Patients’ sera were absorbed with a pool of normal PBMC lysate (n = 5). The 105 kDa and 64 kDa ROR1 bands did not disappear.

Anti-ROR1 antibodies were detected by Western blot in 21 of 23 patients using immunoprecipitated ROR1 proteins. The sera reacted with ROR1 protein bands with the size of 105 kDa and 64 kDa respectively [6,19]. Representative results from 4 non-progressive and 4 progressive CLL patients as well as 4 control donors are shown in Fig 1A. No bands were seen in control donors (n = 20). Non-ROR1 antibodies (anti-CD20 and isotype controls MAbs) were also used for immunoprecipitation of CLL cells. No ROR1 specific bands could be detected (Fig 1B). In anti-CD20 precipitated CLL cells a 37 kDa band was seen when probed with the anti-CD20 MAbs (data not shown). The expected size of CD20 is 37 kDa [20].

Sera from the 21 positive CLL patients were adsorbed with a pool of CLL cell lysates from 10 CLL patients. Anti-ROR1 antibodies against the 105 kDa protein disappeared in 18 patients and decreased in intensity in the remaining 3 patients while antibody against the 64 kDa protein disappeared in 6 and decreased in intensity in 15 patients. Representative blots after adsorption are shown in Fig 1B. Patients’ sera were also absorbed with a pool of normal PBMC. No ROR1 bands disappeared (Fig 1C).

### Detection of anti-ROR1 antibodies using recombinant proteins in ELISA

**Fig 2:**
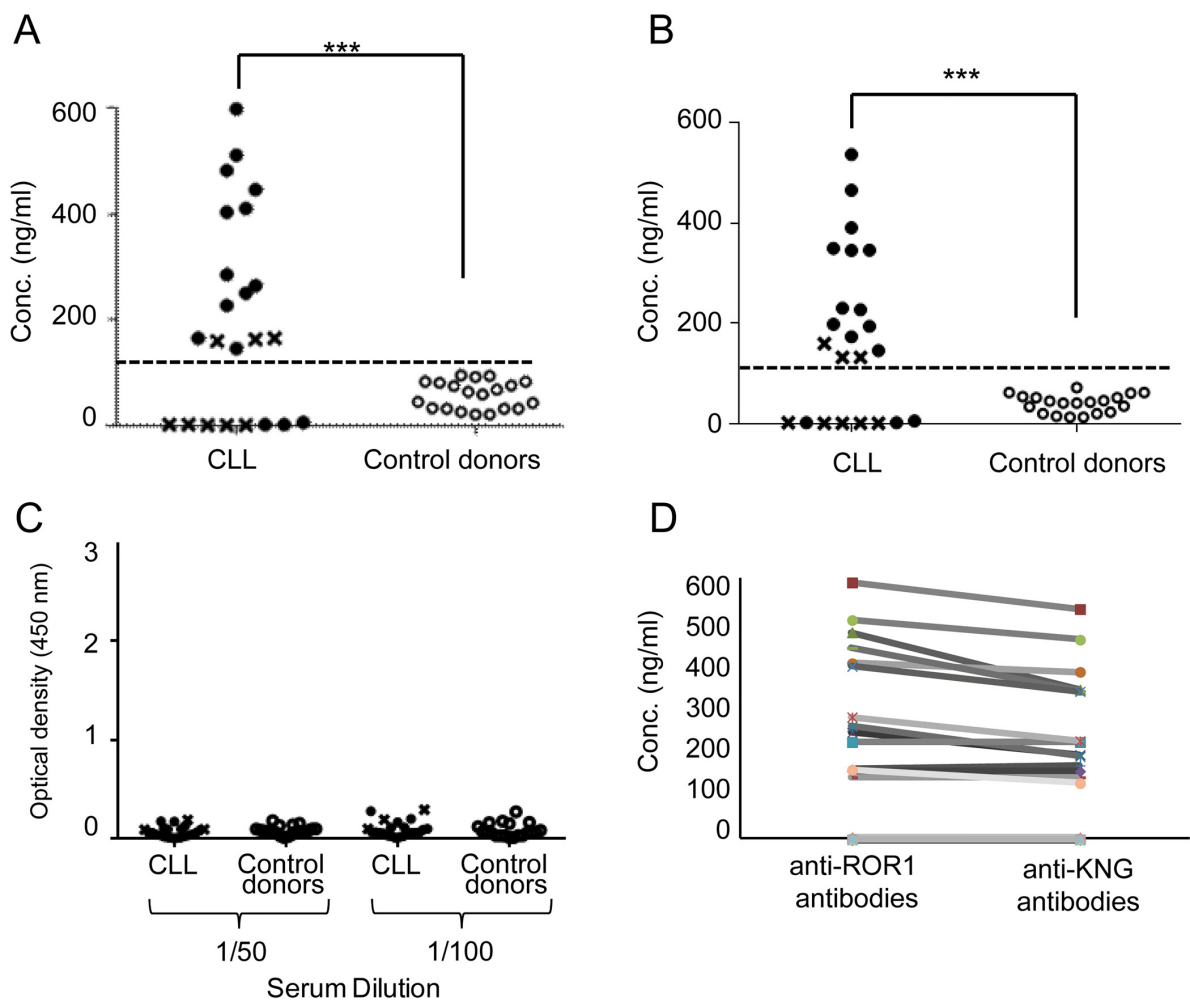
(A) Serum concentrations (ng/ml) of anti-ROR1 antibodies (ELISA) against a full length ROR1 protein and (B) a ROR1 KNG protein in CLL patients (n = 23): (●) non-progressive (n = 15) and (x) progressive (n = 8) CLL patients and (○) control donors (n = 20). Dotted line represents mean+2SD of controls. ***p<0.0001. (C) Reactivity of sera from CLL patients with a recombinant CEA protein. The results are shown as OD values at 450 nm for 1:50 and 1:100 serum dilutions. (D) Relation between conc. of antibodies against a full length ROR1 protein and ROR1 KNG protein in 23 CLL patients.

ROR1 reacting antibodies were also tested in a quantitative ELISA using a recombinant full length ROR1 protein (Fig 2A) and a recombinant ROR1 KNG domain protein (Fig 2B). Sera were considered to contain anti-ROR1 or anti-KNG ROR1 antibodies, if the concentration was above the mean value+2SD of control sera. 15 of the 23 patients had ROR1 antibodies recognizing the full length ROR1 or the KNG domain. The levels of antibody reactivity were significantly higher in CLL patients as compared to controls (p = 0.0001). There was a tendency to a higher frequency of ROR1 antibodies in non-progressive patients (80%) as compared to those with progressive disease (20%) (p = 0.07). None of the sera reacted with a control CEA protein (Fig 2C).

The concentrations of antibodies against the full length ROR1 and the KNG ROR1 proteins resp. of individual patients seemed to be similar (Fig 2D), which may suggest that most of the anti-ROR1 antibodies were directed against the KNG domain.

### Detection of anti-ROR1 antibodies using recombinant proteins in Western blot

**Fig 3:**
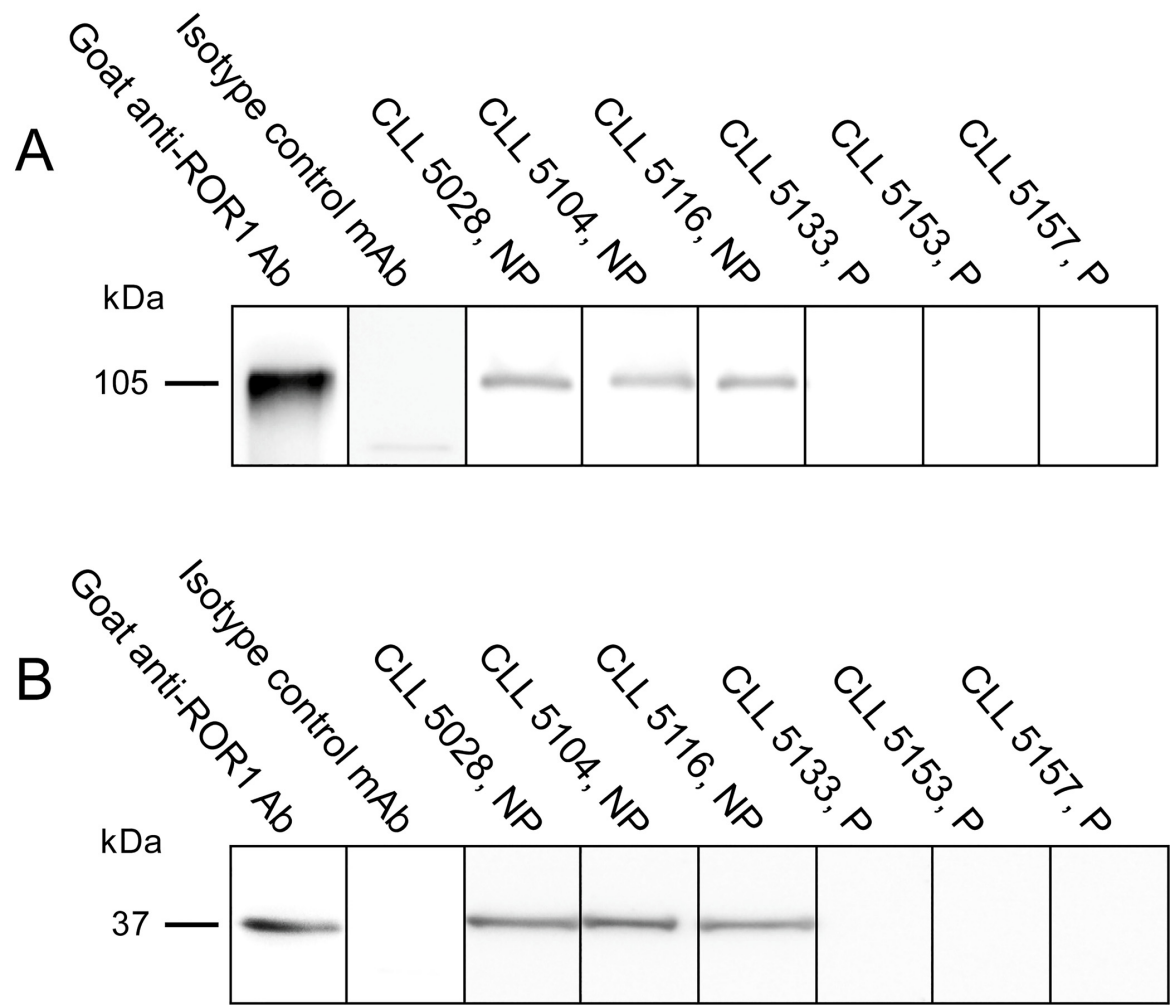
(A) Representative experiments showing antibodies against a recombinant full length ROR1 protein (105 kDa) in 3 non-progressive (NP) and 3 progressive (P) CLL patients. (B) Representative experiments showing antibodies against a ROR1 KNG protein (37 kDa) in 3 non-progressive (NP) and 3 progressive (P) CLL patients. A goat anti-ROR1 antibody and an isotype control MAb served as positive and negative controls respectively.

Anti-ROR1 antibodies were also analysed in Western blot using the recombinant full length ROR1 and KNG proteins. Two bands with a size of 105 and 37 kDa, respectively corresponding to the size of the full length ROR1 and the KNG domain could be detected in five of the 15 patients that were positive in ELISA (Fig 3A and 3B).

### Cytotoxicity of anti-ROR1 antibodies

**Fig 4:**
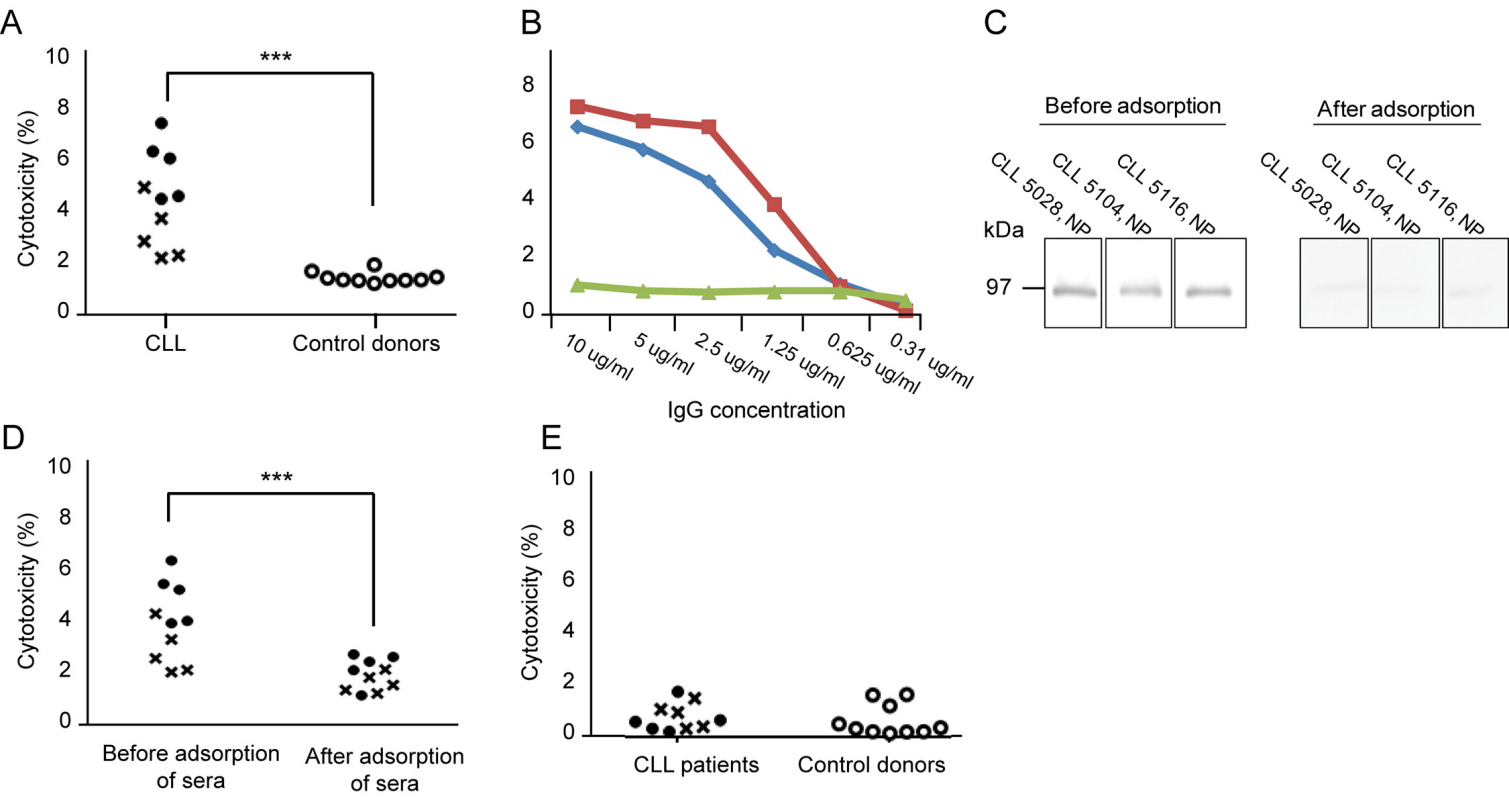
(A) Cytotoxicity (%) of CLL cells (a pool of 10 CLL patients) induced by the IgG fraction alone (10 μg/ml) (without complement or effector cells) from sera of 5 non-progressive (●) and 5 progressive (x) CLL patients and 10 control donors (○). (B) Cytotoxicity (%) of diluted sera of CLL patients 5104 and 5028 (red and blue lines respectively) and pooled IgG of five control donors (green lines). (C) Representative experiments showing antibodies against a recombinant full length ROR1 protein (105 kDa) in 3 non-progressive (NP) CLL patients before and after adsorption of anti-ROR1 antibodies using a pool of CLL cell lysate (n = 10). (D) Cytotoxicity (%) of CLL cells induced by the IgG fraction (10 μg/ml) before and after adsorption of anti-ROR1 antibodies in 5 non-progressive (●) and 5 progressive (x) CLL patients. (E) Cytotoxicity (%) of normal PBMC (a pool of 10 normal donors) induced by the IgG fraction alone (10 μg/ml) from 5 non-progressive (●) and 5 progressive (x) CLL patients as well as 10 control donors (○). P-values (asterics) refer to comparison between CLL and control donors. *p<0.05, ***p<0.0001.

IgG was isolated from 10 CLL patients with the highest concentration of anti-ROR1 antibodies in ELISA and tested for cytotoxicity against CLL cells and normal PBMC using the MTT assay. CLL sera were cytotoxic for leukemic cells but not control IgG (p = 0.0001) (Fig 4A). A dose dependent cytotoxic response of the isolated IgG from CLL patients could be noted (Fig 4B). After adsorption of the sera with the recombinant full length ROR1 protein, ROR1 bands disappeared in Western blot (Fig 4C) as well as the cytotoxic effects of the sera on CLL cells (Fig 4D). There were no cytotoxic effects of sera from CLL patients or control donors using normal PBMC as target cells (Fig 4E).

### Relation between clinical prognostic factors and anti-ROR1 antibody titers

Anti-ROR1 antibody titers were correlated with clinical prognostic factors. A tendency was found for higher titers in Rai stage 0-I compared to II-IV (p = 0.09), but otherwise no differences were noted comparing patients above or below 60 years of age, untreated vs previously treated, white blood cell count below or above 50 x 10^9^/L, or different karyotypes (FISH profiling) (data not shown). However, the number in each subgroup was small.

### T-cell responses to ROR1 peptides

**Fig 5:**
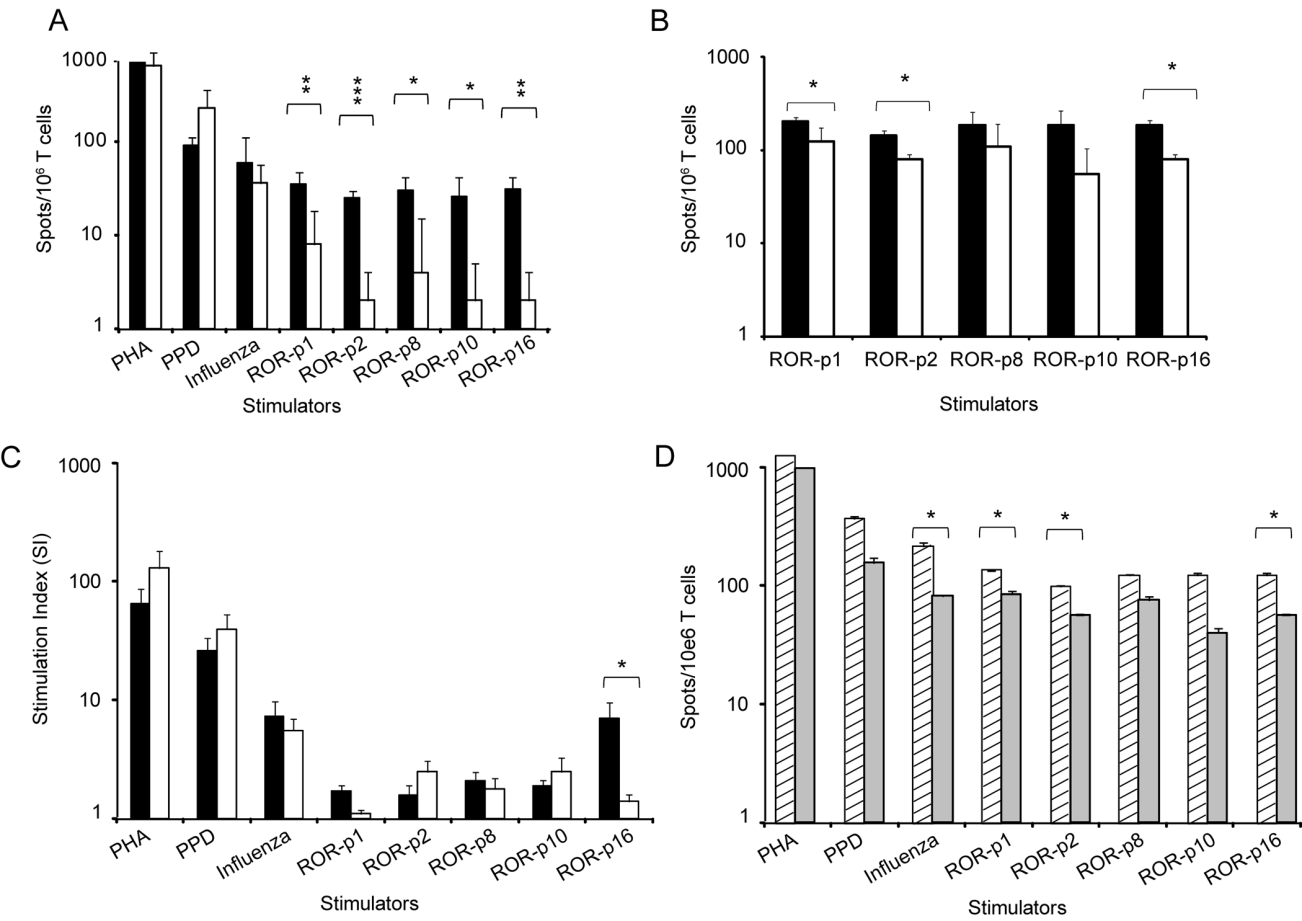
Frequency of T cells (spots/10^6^ T cells) (mean + SEM) secreting (A) IFN-γ and (B) IL-17A (ELISPOT) and (C) stimulation index (SI) (proliferation) (mean + SEM) in response to PHA, PPD and autologous DC loaded with ROR1 derived peptides (p1, p2, p8, p10, p16) or an influenza peptide. (■) CLL patients (n = 9). (□) control donors (n = 6). (D) Frequency of IFN-γ T cells (spots/10^6^ T cells) (ELISPOT) (mean + SEM) in non-progressive (n = 6) (▨) and progressive (n = 3) (■) patients. Background values i.e. number of spots as well as SI after stimulation with an HIV (9 aa) and a Ras (16 aa) peptide were deducted in each experiment. *p<0.05, **p<0.001, ***p<0.0001.

T cell responses against ROR1 peptides were compared in HLA-A2^+^ CLL patients (n = 9) and HLA-A2^+^ control donors (n = 6). A significantly higher frequency of IFN-γ producing T cells after stimulation with each of the four 9 aa long ROR1 peptides as well as the 16 aa peptide was noted in patients as compared to controls (Fig 5A). A weak but significant IL-17A T cell response was also observed against the ROR-p2 and ROR-p8 peptides respectively in patients compared to controls (p<0.05) (Fig 5B). No difference in the frequency of IL-5 secreting T cells could be seen comparing patients and controls (data not shown). There was a significantly higher proliferative response to the 16 aa long peptide (ROR-p16) comparing patients and controls (p<0.05) but not for the other peptides (Fig 5C). The frequency of T cells producing IFN-γ in response to three ROR1 peptides was significantly higher in non-progressive (n = 6) than in progressive (n = 3) disease (p<0.05) (Fig 5D). No statistically significant difference in the frequency of IFN-γ, IL-5 and IL-17A secreting T cells and the proliferative response to PHA and the recall antigens, PPD and influenza was noted comparing patients and controls, but a better IFN-γ response to the influenza antigen was seen in non-progressive compared to progressive patients (Fig 5D).

## Discussion

During early development, the receptor tyrosine kinase ROR1 is of importance for embryogenesis and organogenesis [21], but is then down-regulated and lost or expressed at very low levels in normal adult cells [8,22]. However, ROR1 is expressed in several malignancies including CLL [6] and ROR1 has been considered a tumor associated antigen (TAA) belonging to the group of onco-fetal antigens [6] which might be recognized by the patient’s immune system.

In the present study, we could for the first time show in CLL patients the presence of a spontaneous humoral and T cell response recognizing ROR1. The majority of the patients had an antibody response against immunoprecipitated ROR1 derived from CLL cells. A protein band of 105 kDa was recognized which might represent the full length ROR1 as well as a 64 kDa protein which might represent a cytoplasmic ROR1 isoform [6,19]. A somewhat lower frequency of patients had an antibody response against a recombinant full length ROR1 protein. The difference might be explained by structural differences between the ROR1 protein products. Patients with a humoral response against the full length ROR1 reacted also with a recombinant ROR1 KNG protein indicating that KNG might be an immunodominant domain. Sera containing ROR1 antibodies were cytotoxic for CLL cells. Spontaneously induced antibody responses in patients are most likely polyclonal and of low avidity and titers which might explain the low cytotoxic capability of induced antibodies compared to monoclonal antibodies selected for high cytotoxic capability [13]. An antibody response was most frequently seen in patients with non-progressive disease. The source of the ROR1 antigen preparations, analytical techniques and patient populations may explain the difference in the frequency of patients mounting an antibody response between our study and previous reports [10,23].

In addition to the presence of a humoral response, a ROR1 T cell response (IFN-γ and IL-17A) could be noted. A stronger IFN-γ response was seen in non-progressive as compared to progressive disease similar to the findings for a humoral response. The data may indicate that patients with indolent disease might have a better preserved immune system as revealed by a better response to recall antigen than those with a progressive stage which is in line with previous reports on immune responses against antigens in CLL [24–27].

CLL cells circulating in the blood and lymph nodes have a close contact with T cells and leukemic B cells might be able to present ROR1 peptides to T cells inducing an immune response. CLL cells also continuously undergo apoptosis in e.g. lymph nodes [28] and apoptotic cells might be engulfed by dendritic cells in the microenvironment presenting ROR1 peptides to the immune system. Further promoting the induction of ROR1 immunity.

Positive and negative selections during lymphocyte development induce self-tolerance preventing the immune system to react with self-antigens [29]. The presence of T and B cells against ROR1 in CLL patients might indicate a lack of self-tolerance against ROR1 in CLL. A humoral immune response against ROR1 was also noted in CLL patients vaccinated with Ad-CD154 transduced CLL cells. The induced anti-ROR1 antibodies were capable of blocking the interaction between the ROR1 receptor and the ligand Wnt5a as well as activate complement and immune effector cells to lyse CLL cells [10]. Moreover, CLL patients treated with the immune-modulating drug lenalidomide mounted an anti-ROR1 antibody response [23]. Together with our results these findings may suggest that ROR1 might be an immunogenic TAA. Our findings that induced antibodies seemed to preferentially recognize the KNG domain, a region close to the cell membrane, might be of interest as such antibodies may be of preference for antibody mediated cytolysis [30,31]. Furthermore CD8^+^ T cells engineered to express a ROR1-specific chimeric antigen receptor (CAR) were also able to recognize and lyse primary B-CLL cells, but not normal B cells *in vitro* [32].

Spontaneous immune responses in cancer patients against TAAs have been reported in several studies [33,34]. In multiple myeloma antibodies against cancer-testis antigens were noted in 8% of the patients and a Th1 cell response could also be detected [35]. OVA66, a TAA in ovarian cancer, induced a spontaneous humoral response in 22% of the patients as well as a CTL response [36]. In lung carcinoma, a spontaneous anti-NY-ESO-1 antibody response was noted in 10% [37] and in multiple myeloma in 33% [38] of the patients. In another study, 50% of patients with NY-ESO-1-positive tumors had anti-NY-ESO-1 antibodies [39]. The difference in frequency of patients mounting a spontaneous antibody response might be due to a varying degree of immune-suppression or antigens being more or less immunogenic etc. Interestingly, patients with a spontaneous antibody response against an antigen seemed to have a better capability to mount an immune response against a cancer vaccine [40,41].

We have previously demonstrated that anti-ROR1 monoclonal antibodies were able to induce apoptosis of leukemic CLL cells *in vitro* [13]. Treatment of ROR1xTc-1 transgenic mice using different anti-ROR1 monoclonal antibodies impaired engraftment of ROR1^+^ x Tcl-1 leukemic cells, down-regulated the expression of ROR1 and reduced AKT phosphorylation. The effect seemed to be epitope dependent [42]. Interestingly AKT seemed to be involved in ROR1 down-stream signaling [6,43]. An induced antibody response against ROR1 also seemed to have clinical effects in vivo. ROR1 transgenic mice immunized with a ROR1 peptide developed high-titers of anti-ROR1 antibodies inhibiting engraftment of human ROR1^+^ primary CLL cells [11].

In conclusion, ROR1 may spontaneously elicit a humoral and Th1 T cell response in CLL patients supporting the notion that ROR1 is an immunogenic onco-fetal antigen. Structural and functional characterization of spontaneously induced ROR1 antibodies and T cells in patients may facilitate the development of ROR1 based immunotherapies. Our results and those of others indicate that ROR1 may be an interesting target for cancer immunotherapy.
